# Applications of Smart Textiles for Ambulatory Electrocardiogram Monitoring: Scoping Review of the Literature

**DOI:** 10.2196/74261

**Published:** 2026-03-02

**Authors:** Clarissa Pedrini Schuch, Gabriela Chaves, Bastien Moineau, Sarah Bennett, Meysam Pirbaglou, Edwin Martin Lobo, Milad Alizadeh-Meghrazi

**Affiliations:** 1 Research and Development, Myant Health Corp, 2660 Speakman Dr, Mississauga, ON, L5K 2L1, Canada, 1 4379993141; 2 Institute of Health Policy, Management and Evaluation, Dalla Lana School of Public Health, University of Toronto, Toronto, ON, Canada; 3 Software Quality Assurance, Myant Health Corp, Mississauga, ON, Canada

**Keywords:** cardiac monitoring, smart textiles, electrocardiogram, ECG, remote patient monitoring, wearable, Preferred Reporting Items for Systematic Reviews and Meta-Analyses, PRISMA

## Abstract

**Background:**

Smart textiles (ie, electronic textiles) offer a promising solution to ease continuous electrocardiogram (ECG) monitoring, but their real-world clinical application has been limited.

**Objective:**

This review comprehensively examines the current state of research on textile-based ECG monitoring systems, synthesizing current evidence with respect to performance (ie, signal quality, function under static and dynamic conditions), user experience, and current challenges.

**Methods:**

A systematic literature search across the PubMed, MEDLINE, and Embase databases from 2000 to 2025 identified 34 research papers eligible for inclusion.

**Results:**

Textile-based ECG electrodes demonstrated good signal quality and comfort, particularly under static conditions. Nonetheless, integration into clinical practice requires addressing critical issues, which include greater efforts at validating these technologies in clinical settings and populations, as well as ensuring data security, cost‑effectiveness, user‑friendliness, and data interoperability.

**Conclusions:**

Considering the prominence of feasibility research, the successful clinical integration of textile-based ECG monitoring systems requires comprehensive efforts at establishing a clinical evaluation research base (via clinical trials) and developing regulatory policies.

## Introduction

Cardiovascular disease is a leading cause of morbidity and mortality globally, necessitating the development of improved diagnostic and management tools. In recent years, driven by advances in wearable sensors, wireless connectivity, and cloud computing, there have been substantial developments in large-scale ambulatory collection, transmission, and monitoring of various physiological and clinical outcomes [1-3]. As integrated in telemedicine and remote patient monitoring solutions, these advances offer significant improvements to cardiovascular disease care, where continuous monitoring is crucial for early disease detection and timely intervention [1,2]. Among wearable technologies, electronic textile sensors have emerged as a promising solution for unobtrusive, continuous physiological monitoring in free-living environments [3,4], including in ambulatory electrocardiogram (ECG) monitoring [5,6].

ECG monitoring relies on the detection of the heart’s electrical activity via surface electrodes (placed on the skin), and subsequent transmission of the signal for analysis [7]. Long-term ECG monitoring is vital for the early detection of cardiac arrhythmias, enabling the prompt treatment and prevention of severe complications. Advanced textile–based ECG systems, often termed “smart clothing” or “smart textiles,” integrate sensors and electronics within wearable garments [8-10]. These systems offer the potential for continuous ECG monitoring using dry electrodes, which are embedded in conductive fabrics or textiles [11]. This approach avoids the discomfort and limitations of conventional gel (Ag or AgCl) electrodes and conductive gels [11,12], providing a convenient method for long-term monitoring [5,11,13]. Smart textiles can additionally reduce the need for invasive monitoring modalities (eg, loop and event monitors) and provide a better diagnostic yield (due to the ease of wearing).

Despite enthusiasm, however, adoption of textile-based ECG monitoring in clinical practice remains limited. While extensive research supports the technology’s technical feasibility in measuring ECG [5,6,10], few clinical studies have assessed the validity of this technology in real‑world settings [14]. Challenges hindering widespread clinical adoption include the lack of rigorous clinical validation research and compliance with stringent regulatory requirements (eg, data privacy and security, data interoperability with electronic health records [EHRs]). It is important to acknowledge that the successful translation of textile‑based ECG monitoring from research prototypes to widely adopted clinical and consumer applications also hinges on the development of user‑friendly technologies and user acceptance. On the technical front, maintaining adequate electrode‑skin contact pressure for reliable signal acquisition and ensuring the preservation of garment integrity and performance through repeated washing cycles are critical considerations. While addressing these aspects is not the primary focus of this review, technical developments represent active areas of research, closely linked with user comfort and the long‑term viability of textile‑based ECG systems. This review aims to map and synthesize the existing scientific literature on textile‑based ECG monitoring systems, as integrated in clothing and garments.

## Methods

This review aims to map the scientific literature on textile-based ECG monitoring systems integrated into clothing and garments. Research studies, published in the English language between 2000 and 2025, that investigated the use of textile‑based wearable technologies for ambulatory ECG monitoring in humans were included in this scoping review. Studies were excluded if they focused solely on nontextile wearables. The search time frame (2000-2025) was chosen to capture recent advances in smart textiles, excluding outdated technologies. The language restriction was applied to ensure feasibility and maintain the consistency of data interpretation. The results were reported according to the PRISMA (Preferred Reporting Items for Systematic Reviews and Meta-Analyses) checklist (Checklist 1).

The search strategy used a combination of keywords and controlled vocabulary terms (MeSH and Emtree) across PubMed, MEDLINE, and EMBASE (via OVID) databases. The details of the search are described in Multimedia Appendix 1. Key terms included “Smart textile,” “e‑textile,” “smart clothing,” “textile electrode,” “wearable technology,” “biosensors,” “cardiac rehabilitation,” “remote monitoring,” “ambulatory monitoring,” “Holter,” “cardiovascular diseases,” “arrhythmia,” “atrial fibrillation,” “heart disease,” and “cardiac disorders.” Filters for human studies and publication year limits were subsequently applied to ensure adherence with the specified inclusion criteria.

**Figure 1.**
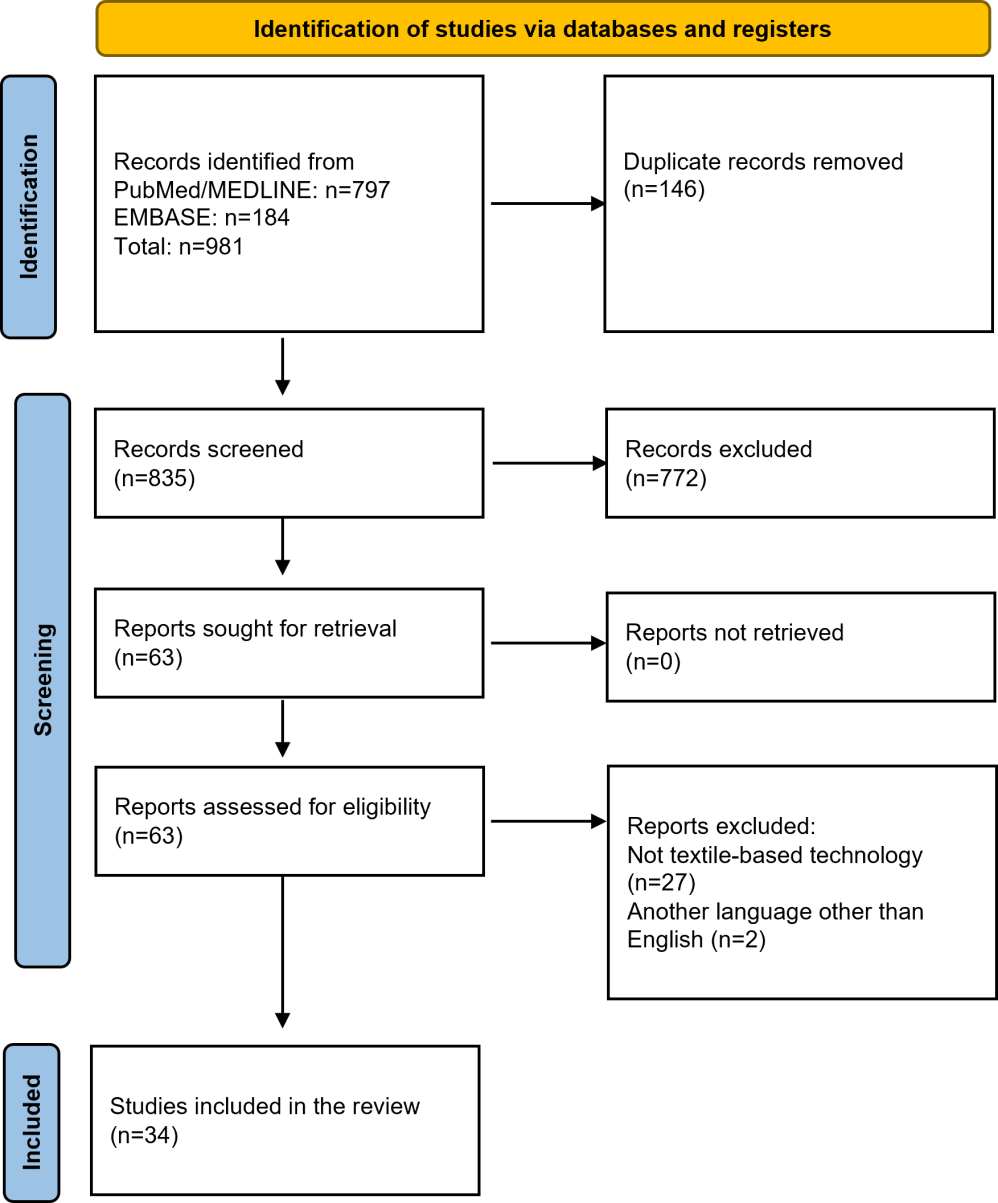
Flowower diagram of search strategy.

The selection process involved 3 stages. First, all identified records were checked for duplicate publications. Second, following the removal of duplicate records, titles and abstracts of the remaining records were screened for relevance to the research question and the eligibility criteria. Third, full‑text papers of potentially relevant studies were reviewed to confirm eligibility. Any discrepancies were resolved through discussion among the review team members. This process ensured a rigorous selection of studies appropriate for inclusion in the scoping review. In total, across 981 records identified across databases, 63 records were selected for full‑text review of eligibility. Following this review, 34 studies were selected for inclusion (Figure 1).

Given the limited number of published randomized clinical trials focusing on textile-based ECG in real-world clinical settings, this scoping review included feasibility studies, with a focus on ECG signal quality and user comfort as key outcome measures. This methodological choice acknowledges potential limitations in generalizability. It emphasizes the need for further research that incorporates diverse populations and real-world conditions to fully evaluate the clinical utility of this technology. The limited number of studies reporting on clinical outcomes directly reflects the current state of research in this area. The 34 studies were then comprehensively synthesized with respect to 3 key areas of research in smart textile–based ECG monitoring:

1. Comparative signal quality: To evaluate the signal quality of smart textile electrodes to traditional gel electrodes in evaluating various ECG indices (ie, QRS complex, P-wave, T-wave, R-peak).
2. Performance in static and dynamic conditions: To evaluate the performance of textile electrodes under static (eg, resting) and dynamic (eg, movement) conditions, examining the impact of motion artifacts on signal quality.
3. User experience: To evaluate user comfort, which is a critical factor for long-term adoption and use of smart textile–based ECG monitoring platforms, considering factors such as fabric type, electrode placement, and overall wearability.

Finally, this review outlines a roadmap for key clinical research priorities, together with highlighting the challenges that hinder the wider adoption and integration of textile‑based ECG monitoring systems.

## Results

### Research Landscape

Between 2000 and 2025, smart textile technologies for ECG monitoring have seen steady progress, with research efforts predominantly based on prototype development [15-33] and observational research studies [34-44]. Smart textile technologies reviewed here use a range of conductive materials (eg, silver, copper, stainless steel, and metal inks), as incorporated into different garment types (eg, knitted patches, bands, vests, t-shirts, and bras). Among key design challenges for e‑textiles is reliable electrode adhesion, particularly given the contrasting properties of metallic electrodes and the nonmetallic nature of clothing. To address this, various integration techniques are used, including direct knitting or weaving of electrodes into the fabric, embroidery of conductive yarns, adhesive bonding with specialized durable adhesives, or printing with conductive inks requiring encapsulation. Garment design, such as the use of compression garments, and material selection, prioritizing rough electrode surfaces for improved mechanical interlocking, also play crucial roles. Different electrode materials and garment designs influence the contact pressure between the electrode and the skin. Contact pressure has an important role in ensuring the proper placement of the wearable and providing comfort. Excessive pressure can improve signal quality but can also increase discomfort and cause skin damage, while insufficient pressure can compromise the device’s functionality [40]. Some studies have investigated compression garments to enhance contact pressure, while others have focused on optimizing electrode design to distribute pressure evenly. While these techniques offer promising solutions, the long-term durability of these adhesion methods and their resistance to repeated washings remains a key focus for future research. Wash durability is a critical consideration for textile-based ECG systems. Repeated washing cycles can degrade conductive materials, alter garment shape, and compromise electrode-skin contact. Research is ongoing to identify durable and washable electrode materials, as well as garment construction techniques that minimize performance degradation over time [45].

Although recent reviews, such as that by Xu et al [46], have explored the landscape of smart textiles in sports and health care, this work distinguishes itself by providing a focused analysis of textile-based systems specifically engineered for ECG monitoring, with a particular emphasis on clinical translation and utility. As evidenced by the analysis in Table 1 (static conditions) and Table 2 (dynamic conditions), the selection of electrode type significantly influences the performance and applicability of these systems. This segmentation by static and dynamic conditions is crucial because static conditions provide a controlled environment to assess basic electrode performance, while dynamic conditions, which introduce motion artifacts and other real-world challenges, are essential for evaluating the true utility of these systems. For example, silver-coated yarns are widely used in static settings, but their susceptibility to motion artifacts can be a limiting factor in dynamic environments. Novel materials, such as conductive polymers, hold promise for improved flexibility and signal stability, though further research is needed to validate their long-term performance in both static and dynamic scenarios. This highlights the importance of carefully considering the intended application and monitoring environment when selecting the optimal electrode technology for textile-based ECG monitoring.

**Table 1.** Studies summarized by static monitoring setting.

Author	Study type	Sample size	Electrode type	Findings	Condition
Mestrovic et al [26] (2007)	Observational	31	Textile-based piezoresistive transducer (vest)	Readable signals in cardiac inpatients lying	Static
Vojtech et al [30] (2013)	Prototype or concept	1	Silver-coated polyester and cotton nanoparticles	The amplitude of the individual waves of ECG signal obtained using the textile electrodes was smaller than the reference signal	Static
Dai et al [21] (2016)	Prototype or concept	6	Flexible polypyrrole textile electrodes	Heart rate accuracy of each subject was more than 95%	Static
Kannaian et al [29] (2012)	Prototype or concept	3	Embroidered the conductive yarn on polyester fabric	Electrocardiogram signal from the textile electrode was similar to the gelled electrode	Static
Pandian et al [20] (2008)	Prototype or concept	25	Sensors (silicon rubber with pure silver fillings)	RR^a^ intervals presented good stability	Static
Tsukada et al [36] (2019)	Observational	66	T-shirts for men and brassieres for women with textile electrodes	P-wave, QRS, and T-wave were comparable between the textile and conventional electrodes	Static
Nigusse et al [19] (2020)	Prototype or concept	6	Silver-printed textile electrodes	The signals collected by silver-printed textile electrodes showed clear R-peaks without missing and false peaks, indicating good waveforms	Static
Arquilla et al [35] (2020)	Observational	8	Textile electrodes (silver thread) were sewn in a chest-mounted configuration	The sewn electrodes produced clean data, allowing the measurement of beat-to-beat variability and average heart rate	Static
Fouassier et al [25] (2020)	Feasibility or pilot	30	Smart t-shirt composed of 13 textiles electrodes made of silver yarns and hydrogel pads that released water vapor	A sinus rhythm was distinguished for all recordings obtained with both smart textile and reference system for all 3 static conditions	Static

a RR: R-R interval.

**Table 2.** Studies summarized by dynamic monitoring setting^a^.

Author	Study type	Sample size	Electrode type	Findings	Condition
Hsu et al [24] (2019)	Prototype or concept	3	Noncontact dry electrodes in an elastic chest vest and attached with Velcros (not embedded)	ECG signal quality obtained by the noncontact electrode was similar to the conventional gel electrode. ECG signal quality was also good while walking.	Dynamic
Paradiso et al [15] (2005)	Prototype or concept	1	Fabric with strain sensors	Metal-based fabric electrodes reliably recorded bioelectric potentials.	Dynamic
Perez de Isla et al [39] (2011)	Feasibility or pilot	31	Noncontact dry electrodes in an elastic chest vest	Good intermethod agreement for common ECG parameters. Nuubo shirt allowed pretest and posttest echocardiography. The system offers continuous, noninvasive, remote monitoring. The initial device had frequency limitations (0.5-100 Hz), affecting ST-segment analysis.	Dynamic
Trindade et al [17] (2016)	Prototype or concept	5	T-shirts comprised of 5 skin-contact textile electrodes	T-shirt prototypes provided adequate performance in standing states. Motion artifact interference, mainly caused by friction between the textile electrodes and the skin, considerably limited the performance of the prototypes in mobile contexts.	Dynamic
Pola et al [27] (2007)	Prototype or concept	4	Silver electrodes	Jogging caused the biggest problem in measurements.	Dynamic
Gonzales et al [31] (2015)	Prototype or concept	Not reported	T-shirt with 3 dry silver-based electrodes	Good efficacy of dry electrodes even in a high-motion environment.	Dynamic
Li et al [18] (2020)	Prototype or concept	1	Textile electrodes (silver-coated nylon yarns) knitted into a T-shirt	The smart clothing showed good performance for measuring ECG signals.	Dynamic
Ghahjaverstan et al [43] (2023)	Feasibility pilot	20	Dry electrodes made from conductive yarns, knitted within an elastic chest band	HR^b^ measurements comparable to the reference system across all tasks, including exercising.	Dynamic
Romagnoli et al [47] (2014)	Observational	12	Smart textile system (GOW) with embedded textile electrodes in shirt, transmitting data to an electronic module	Excellent agreement between GOW and ECG for RR^c^ intervals (LoAs^d^ around ±3 ms). Good agreement for MeanRR, SDNN^e^, SD2. Poor agreement for RMSSD^f^, HF^g^, LF/HF^h^, HFnu^i^, and SD1 (wide limits of agreement). The GOW system showed overall good accuracy for HR but limited accuracy for many HRV^j^ parameters.	Dynamic
Coyle et al [38] (2010)	Prototype or concept	Not reported	Piezoresistive sensor	ECG signals gathered during rest showed high quality.	Dynamic
Di Rienzo et al [16,41] (2005, 2006)	Prototype or concept; observational (development phases for the same system)	1; 31	Textile-based piezoresistive transducer (vest)	Readable signals in cardiac inpatients pedaling.	Dynamic
Di Rienzo et al [48] (2013)	Observational	40	Textile vest with knitted conductive‑fiber electrodes (single‑lead ECG)	Readable ECG 95.9% vs 82.7% (traditional); artifact rate 4.1% vs 17.3%; arrhythmia detection sensitivity 99.7%, specificity 99.9%; PQ/QRS comparable; RR interval error ~1 ms	Dynamic
Weder et al [32] (2015)	Prototype or concept	12	Two Ag- or Ti-coated polyethylene terephthalate electrodes embedded into a chest belt	The embroidered electrodes presented signals comparable with Ag or AgCl gel electrodes	Dynamic
Pani et al [33] (2016)	Prototype or concept	10	Patch of textile electrodes made by treating conventional fabrics with a highly conductive solution of PEDOT:PSS	Textile electrodes showed similar performance, or even better, in wet conditions	Dynamic
Alizadeh Meghrazi et al [13] (2020)	Prototype or concept	6	Silver-plated nylon yarns and carbon-coated nylon yarns knitted in a waistband	ECG signals were reliably obtained from different locations on the waist	Dynamic
Fukuma et al [49] (2019)	Feasibility or pilot	100	Textile electrodes (Hitoe fabric with PEDOT-PSS) embedded in a t-shirt	The system was suitable for lifestyle or sports activities, demonstrating AF^k^-detection performance similar to that of other wearable devices	Dynamic
Pagola et al [50] (2023)	Feasibility or pilot	163	Textile electrodes in a garment	The textile wearable Holter monitoring detected pAF^l^ in 35.37% of patients	Dynamic
Teferra et al [34] (2021)	Feasibility or pilot	1	Textile electrodes embedded in a smart ECG vest	Performance was comparable to a traditional 3-lead Holter monitor	Dynamic
Machino et al [42] (2023)	Randomized controlled trial	67	Dry textile electrodes (PEDOT-PSS and nanofiber) embedded in a garment	Garment ECG (2-week) detected AF recurrence in 18% of patients, significantly higher than 24-hour Holter (6%)	Dynamic
Amami et al [44] (2022)	Feasibility or pilot	31	Silver textile electrodes embedded in an undershirt	Wearable undershirt electrode demonstrated abilities comparable to Holter ECG for ECG monitoring and arrhythmia detection	Dynamic
Olmos et al [51] (2014)	Observational	31	nECG SHIRT: Biomedical shirt with integrated textile electrodes (BlendFix) for ECG signal acquisition	Excellent correlation between Nuubo system and conventional tilt table test for commonly assessed ECG parameters during tilt testing	Dynamic
Yu et al [52] (2017)	Cohort study	5	Wearable 12-lead ECG T-shirt with 10 dry textile electrode patches (Shieldex Medtex P180) and active electrodes	Average per-lead signal coverage ranged from 20.9% to 56.3%. After combining data from all leads (temporal fusion), overall coverage improved to up to 81.9%. A 3-stage artifact detection algorithm effectively identified artifacts from 50-Hz noise and motion.	Dynamic
Pagola et al [22] (2018)	Feasibility or pilot	146	Three-lead ECG vest and 1-lead ECG chest band	Both garments were similarly comfortable during the day and night. However, the vest group presented a longer time of compliance and time analyzed than the chest band. The percentage of missed signal was lower in the vest group. The rate of undiagnosed AF detected with textile Holter was 21.9%	Dynamic
Steinberg et al [23] (2019)	Prototype or concept	15	Bra or shirt with integrated sensors recording a single-lead ECG	The wearable ECG sensor's signal quality and accuracy were equivalent to Holter monitoring. Signal coverage of R-R intervals showed a very close overlay between the wearable sensor and Holter signals. The wearable sensor presented high wearing comfort and minimal risk of skin irritation.	Dynamic
Neri et al [53] (2024)	Observational	30	Crop top garment with embedded polymer–based electrodes	YouCare System showed 70% “Good,” 12% “Acceptable,” and 18% “Not Readable” ECG signals	Dynamic

a Prototype or concept: Early engineering or technical validation studies, often with small samples or bench testing. Feasibility or pilot: Preliminary human studies assessing usability, signal quality, or performance in real-world conditions. Clinical studies: Observational, cohort, or randomized controlled designs.

b HR: heart rate.

c RR: R-R interval.

d LoA: limits of agreement.

e SDNN: standard deviation of NN intervals.

f RMSSD: root mean square of successive differences.

g HF: high-frequency component.

h LF/HF: low-frequency/high-frequency ratio.

i HFnu: normalized high-frequency component.

j HRV: heart rate variability.

k AF: atrial fibrillation.

l pAF: paroxysmal atrial fibrillation.

### Comparative Signal Quality of Textile-Based ECG Monitoring and Standard Gel Electrodes

The standard ECG signal comprises morphological features (ie, P-wave, QRS, and T-wave), reflecting atrial and ventricular depolarization and repolarization, and temporal features (ie, PR, QRS, QT, and R-R intervals), representing durations between these events [5]. These features provide key insights into cardiovascular physiology and pathology, making continuous long-term ECG monitoring essential for detecting cardiovascular disorders and abnormalities [54].

Traditionally evaluated over the long term using Holter monitors, ambulatory ECG monitoring relies on adhesive gel (Ag or AgCl) electrodes, which provide excellent signal quality [24,35]. However, long-term use is often limited by skin irritation, gel drying, and electrode detachment due to perspiration [24,35,55]. Smart textile electrodes offer a potential solution to these issues by using conductive fabrics, materials, or metals to acquire ECG signals without the need for gels [11].

The demonstration of the accuracy and reliability of smart textile–based ECG monitoring platforms is essential to enhance their adoption in clinical settings. In relation to identifying morphological and temporal ECG features, most studies have focused on the QRS complex, P-wave, T-wave, and R-peak amplitude variations and smart textiles’ signal-to-noise ratio. For example, in an early demonstration of the feasibility of textile-based ECG monitoring, Di Rienzo et al [16,56] used a conductive fiber vest to accurately detect arrhythmias and heart rate compared to a traditional ECG recorder, while Paradiso et al [15] showed comparable results with a metal-yarn vest. Further research investigated various conductive materials (eg, silver-coated nylon, stainless steel yarn [26]), electrode placement (chest bands, t-shirts, wristbands [26,27,29,30]), and different textile constructions, demonstrating good signal quality in many studies in agreement with reference devices [13,19,21,23,26,29,30,35,42-44,47,50,52]. However, some studies reported issues, such as noise from dry skin [27], signal amplitude discrepancies due to electrode placement [30], and waveform irregularities [18]. While many studies focused on signal quality and accuracy, and some incorporated signal processing algorithms to improve data quality [15,16,39,57], few addressed the integration of textile-based ECG data into clinical practice [34,42-44,50,58-61].

Several studies demonstrated good textile-based ECG signal quality [20,21,26,29,32,34,36,38,46,52,57], highlighting the technology’s promise for long-term monitoring. However, limitations remain: data transmission methods (eg, Bluetooth, Wi-Fi) often require extensive postprocessing; most studies were conducted in controlled laboratory settings with healthy participants and did not use fully integrated wearable form factors; and the clinical validation and long-term feasibility remain insufficiently explored. Future research should address these limitations through more comprehensive real-world studies incorporating diverse populations and integrated clinical workflows to ensure reliable and meaningful data.

### Textile-Based ECG Monitoring Performance During Static and Dynamic Conditions

The division of studies based on monitoring setting (static vs dynamic) reveals key trends in the field of textile-based ECG monitoring. As demonstrated in Table 1, 9 of the reviewed studies focused on static conditions, likely reflecting the initial stages of development and validation for these technologies. The rationale for this segmentation lies in the fundamentally different challenges presented by each scenario. The static setting allows for the controlled assessment of signal quality and electrode performance, minimizing the confounding effects of motion artifacts and providing a baseline for evaluating the system’s potential. However, the number of studies in the dynamic category demonstrates a growing interest in the application of these systems in real-world scenarios. This shift introduces new challenges, as the dynamic setting often leads to increased motion artifact and greater variability in electrode-skin contact impedance. Examining the types of activities within the dynamic setting (walking, jogging, cycling, etc) further highlights these challenges, with activities involving more vigorous movement generally resulting in lower signal quality.

The inherent flexibility of textile-based ECG systems introduces challenges related to motion artifacts. Di Rienzo et al [48] explored the impact of dynamic conditions, examining the impact of motion artifacts on signal quality in telemedicine applications. The electrode-skin interface is particularly susceptible to disruption during dynamic movements, leading to noise and signal degradation, especially with dry or hairy skin [11,19,33,37]. Numerous studies have investigated the impact of both static (sitting, lying, standing) and dynamic (eg, walking, jogging, sit-to-stand) activities on ECG signal quality [17,20,24,25,31,32,34,36,38,39,41,52]. For example, Perez de Isla et al [39] evaluated a dynamic ECG monitoring system (Nuubo’s dynamic ECG) against a conventional treadmill–based ECG monitoring system during exercise electrography and found comparable performance between the 2 systems on baseline and peak heart rates. Similar results were also seen by Olmos et al [51] for heart rate and maximum PR interval on the tilt table test between Nuubo’s technology ECG shirt and conventional ECG monitoring.

In relation to other ECG parameters, Di Rienzo et al [48,57] also observed adequate QRS complex detection during walking and pedaling, while Pandian et al [20] reported good consistency in R-R, QRS, and QT intervals during walking with a textile vest. However, Trindade et al [17] noted higher noise amplitude with textile electrodes compared to gel electrodes during walking, and Tsukada et al [36] observed increased motion artifacts with trunk twisting. Studies included various strategies to mitigate motion artifacts, including the optimization of electrode placement, use of compression bands to enhance electrode-skin contact, and application of skin moisturizers or humidification techniques [11,17,33]. The use of a wetting device was also shown to improve signal quality in both static and dynamic conditions [32].

### User Comfort

Wider clinical adoption of textile-based ECG monitoring requires user acceptance, which is linked to comfort and ease of use [11,62]. While high signal quality and accuracy are crucial, a comfortable and unobtrusive system is essential for encouraging prolonged wear and consistent data acquisition [6]. However, user comfort has been under-investigated, with only a few studies addressing this critical aspect as a secondary outcome [22,23,35,44,63].

These studies offer key insights into user perceptions. Pagola et al [22] found that patients with stroke preferred the e-textile vest over a conventional chest band for long-term use, with comparable comfort levels across day and night. Wu et al [62] reported that an e-textile t-shirt was more comfortable than traditional gel electrodes. Neri et al [53], comparing an e-textile–based ECG device to a standard Holter monitor, reported significantly greater patient comfort in the e-textile monitor, compared to the standard monitor. Steinberg et al [23] also noted greater comfort and low skin irritation with e-textile sensors, integrated into bras and shirts during 24-hour monitoring, while Arquilla et al [35] found no significant comfort difference compared to traditional electrodes. Similarly, Montazeri Ghahjaverstan et al [43] reported that all pediatric participants found the textile-based chest band easy to wear, nonirritating, and generally comfortable, with 95% rating the design as appropriate and 75% willing to use it for continuous monitoring at home.

## Discussion

This review highlights the significant progress, as well as the challenges faced in optimizing e-textile–based ECG monitoring. Our findings address pertinent issues related to the optimal design and adoption of textile-based ECG monitoring technologies with respect to signal quality, static and dynamic performance, and user comfort.

### Signal Quality

In relation to signal quality, the reviewed studies primarily evaluated ECG signal quality of e-textiles in healthy participants within controlled laboratory settings. The current proof-of-concept studies and feasibility testing [64,65], though essential for establishing minimum viable products, do not address the complexities of adopting e-textile–based ECG monitoring in clinical practice. Therefore, definitive conclusions about the effective use and integration of e-textiles in clinical practice cannot be drawn. Still, these studies achieved reliable ECG signals in e-textiles compared to standard adhesive electrodes and clarified key requirements to achieve reproducible ECG signals in e-textiles. These included consistent electrode placement, stable contact, supported by optimized electrode design, appropriate garment fit, garment care, and wash durability. Overall, these findings point to the need for evaluations of e-textile–based ECG monitoring in everyday life and clinical settings against standard modalities (ie, Holter monitors), incorporating diverse populations, health conditions, and standardized testing protocols.

### Static and Dynamic Performance

E-textile–based ECG monitoring is particularly susceptible to motion artifacts, which disrupt ECG signals and degrade signal quality. The majority of studies included monitored ECG in static conditions, likely reflecting the initial validation efforts. While these studies indicated equal-to-superior performance for static e-textile–based ECG assessment [20,26,29,30,38,57], fewer studies included dynamic e-textile–based ECG assessment. Despite the lack of standardized protocols, many studies demonstrated the satisfactory detection of QRS complexes and rhythm abnormalities during moderate dynamic activity [20,34,38,52,57]. Nonetheless, motion artifacts significantly impacted signal quality during more strenuous activities [17,25,33,36]. A limitation of the current literature in relation to dynamic e-textile–based ECG assessment is the lack of standardized protocol, making it difficult to directly compare results across studies. Furthermore, studies in realistic, uncontrolled dynamic environments are still relatively scarce, highlighting the need for further research to address the practical challenges of implementing textile-based ECG monitoring in everyday life. In addition to factors that influence signal quality (mentioned previously), further advancements in signal processing and machine learning are needed to reliably extract clinically meaningful data during dynamic conditions [39,60,61].

### User Comfort

Although some feasibility and prototype studies report that textile electrodes may improve comfort compared to traditional gel electrodes [17,21], evidence remains limited and heterogeneous. Independent studies [22,23,35,63] have noted favorable comfort outcomes, while 1 study from our group [43] found the textile band to be “generally comfortable.” These findings should be interpreted with caution given the potential for bias and the small sample sizes involved. Overall, comfort remains an underresearched area, and further independent clinical evidence is needed to confirm these preliminary observations. Future research should prioritize standardized assessment of user experience using validated tools and include diverse populations, as comfort and ease of use are critical for long-term adoption.

### Clinical Integration

The potential for greater use and clinical integration of e-textiles is promising, yet significant challenges remain. These include interoperability with existing EHR systems, clinical validation, patient privacy, cost-effectiveness, timely feedback mechanisms, and user-friendliness. Effective workflow integration also requires clear standards for data review frequency, protocols for urgent findings, and sustainable reimbursement models. Frameworks, such as the ABCD model [58]—which evaluates device accuracy, clinical utility, regulatory approval, and cost—can guide adoption. Importantly, these frameworks must incorporate both clinician and patient perspectives [66-68] to ensure usability and adherence.

Recent clinical investigations provide encouraging evidence of feasibility and diagnostic value in real-world settings. Fukuma et al [49] demonstrated that a t-shirt with PEDOT-PSS electrodes was suitable for lifestyle and sports activities, achieving atrial fibrillation (AF) detection performance comparable to other wearables; although this study was included in our review, it primarily involved asymptomatic participants with cardiovascular risk factors rather than patients with established disease. Pagola et al [50] reported that intensive 90-day textile Holter monitoring detected paroxysmal AF in 35.37% of patients, underscoring the benefit of extended monitoring. Machino et al [42] showed that a 2-week garment ECG detected AF recurrence in 18% of patients—significantly outperforming 24-hour Holter monitoring (6%). Similarly, Amami et al [44] found that an undershirt with silver textile electrodes provided ECG monitoring and arrhythmia detection comparable to conventional Holter devices. Collectively, these findings indicate promising feasibility and signal quality; however, conclusions about clinical robustness cannot be drawn without formal quality assessment and larger trials.

### Recommendations

The rapid evolution of telemedicine and digital health tools presents significant opportunities to improve cardiovascular disease management [69-71]. Smart textiles offer a promising pathway toward enhanced remote patient monitoring, providing benefits for patients, health care providers, and the overall health care system. However, successful integration requires addressing several key aspects.

1. Wearable data acquisition: This pillar focuses on developing robust and reliable wearable systems capable of accurately monitoring, collecting, and preprocessing ECG data from textile-based sensors. This includes considerations, such as sensor design, electrode placement, and signal quality under various conditions.
2. Data transmission and communication: The seamless and secure transmission of ECG data from the wearable device to a remote monitoring center (smartphone or PC) is crucial. This necessitates robust and reliable data communication networks, addressing issues such as signal strength, data security, and bandwidth limitations.
3. Cloud-based data analytics and clinical integration: A powerful cloud-based analytics platform is needed to process, analyze, and interpret ECG data efficiently, providing clinicians with readily accessible, accurate, and clinically relevant information [2,6,9]. This platform must facilitate timely alerts for acute events, support offline data access, and seamlessly integrate with existing EHRs. Data processing and analysis techniques are essential components of this system, requiring sophisticated algorithms to filter noise, identify key patterns, and provide meaningful interpretation [72,73]. The scalability and reliability of the backend infrastructure, including data storage and retrieval, are essential factors determining the system’s performance.

**Figure 2.**
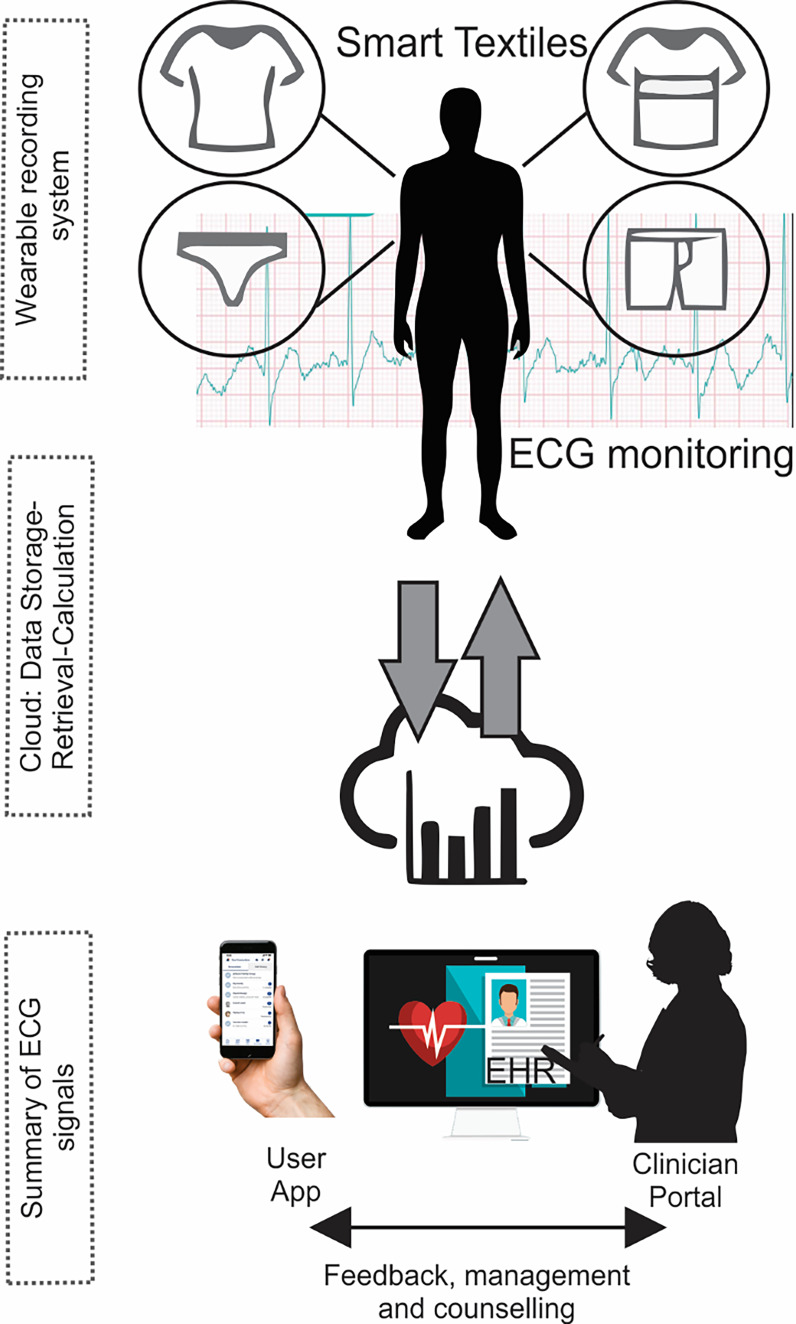
Smart textiles data workflow and integration in clinical practice. Textile-based sensors embedded in clothes or garments continuously measure electrocardiogram (ECG) signals.

Clinicians require user-friendly clinical portals to visualize and manage this data effectively, enabling timely diagnosis and intervention. Patients and caregivers should have access to relevant information for their self-management and remote health monitoring (Figure 2). Finally, user training and education are critical, particularly for individuals with low digital literacy [74]. Addressing these 3 pillars will be key to realizing the full potential of smart textiles in improving cardiovascular care.

### Current Issues and Challenges

**Figure 3.**
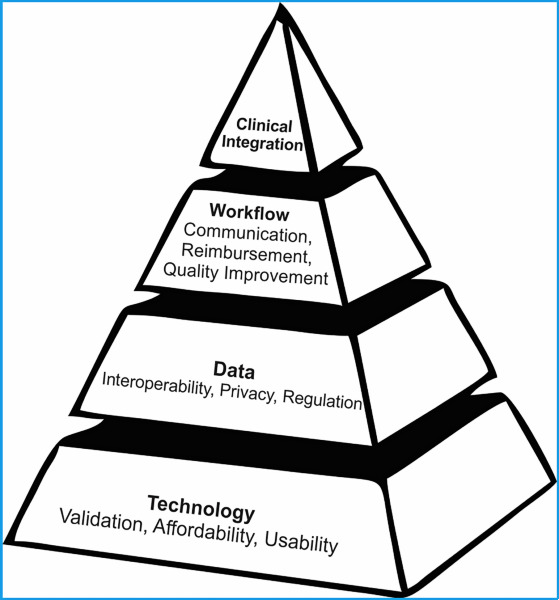
Challenges for smart textile integration into clinical practice.

While textile-based ECG technology shows promise in terms of signal quality, several challenges must be addressed to facilitate its broader adoption in clinical practice. This section outlines key obstacles across 3 interconnected areas: technology, data management, and clinical workflow integration (Figure 3, Table 3).

**Table 3.** Challenges and recommendations for smart textile integration into clinical practice.

Theme	Challenges for clinical integration	Recommendation
Technology	Lack of clinical validation of smart textiles for ECG^a^ monitoring. Poor data validity hampers the repeatability of measurements and impairs interoperability and data exchange. Affordability and cost may limit the uptake of the smart textile for ECG monitoring. Absence of information about user needs, perception, and acceptance of the smart textile for ECG monitoring.	Extensive clinical validation regarding the safety, effectiveness, and performance of the smart textile to monitor physiological data over long periods in free-living conditions. Private insurance companies and government may partially support the cost of smart textiles for ECG monitoring. A comprehensive understanding of users' perceived value, perceived usefulness, perceived ease of use, and satisfaction must be met by health care professionals and users for the widespread adoption of smart textile devices. Surveys, interviews, and focus groups to address the user acceptability issue.
Data	User privacy and security concerns related to the misuse of personal health information during data transfer and real-time data streaming. Inefficient interoperability among devices, applications, systems, and domains. Absence of clear regulatory policies governing smart textile for ECG monitoring.	Clear guidelines providing the privacy, confidentiality, and proper use of electronic medical information. Ensure that users feel comfortable and confident sharing a significant amount of data with data analytic companies, health care providers, and insurance companies. Uniform and straightforward data formats; standard communication protocols for information exchange between smart textiles and other platforms. Adopt devices cleared or approved by regulatory agencies (i.e., FDA^b^). Expansion of HIPAA^c^ policies aligned with remote patient monitoring technologies, such as smart textiles.
Clinical workflow	Efficient processes for communicating actionable or urgent data between health care providers, patients, and caregivers are not yet well defined. In particular, the appropriate frequency of data review and the personnel responsible for monitoring incoming data still need to be established. Without clear workflows, there is a risk of data overload, which can delay timely and appropriate clinical responses. Reimbursement structures are not fully implemented for remote patient monitoring using smart textiles technologies. Challenges using digital health data gathered from electronic health record systems, personal health records, and other systems (eg, smart textile) for clinical quality improvement.	Development of a clinical dashboard for data reporting. Operationalize and triage the data and define realistic response time. Health care providers can review regularly acquired ECGs during in-person or virtual visits. Timely feedback can be guaranteed if the platform allows the health care provider to set customizable notifications, for example, in case an abnormal event is detected. Clear reimbursement models addressing remote monitoring and data review. More studies to assess the costs and cost-effectiveness of remote monitoring are necessary to determine reasonable reimbursement rates. Clear regulations to use the smart textile data, allowing it to be aggregated, linked with other forms of data, and leveraged to generate new knowledge and promote clinical quality improvement.

a ECG: electrocardiogram.

b FDA: Food and Drug Administration.

c HIPAA: Health Insurance Portability and Accountability Act.

### Technological Challenges

Technological challenges include the following:

- Clinical validation: While numerous studies demonstrate the feasibility of textile-based ECG electrodes in terms of signal quality, none have yet achieved routine clinical adoption (technological readiness level 9). A substantial body of clinical data is needed to validate the technology’s fidelity, interoperability, safety, and effectiveness as intended [75].
- Affordability and cost: While smart textiles could potentially reduce health care costs through optimized patient management and early intervention [61], this remains unsubstantiated. The cost-effectiveness of this technology needs further investigation, considering both initial investment and long-term operational costs [76-78]. Reimbursement policies from insurance companies and governments are crucial for broader accessibility [66,79].
- Usability and acceptability: Limited research exists on user perceptions and acceptance of smart textile ECG monitoring. Factors, such as comfort (thermal, tactile, style, pressure), ease of use, perceived value, and the ability to share data with health care providers, significantly influence user adoption [66,67,69,80,81]. Further research using user-centered design methodologies (surveys, focus groups, interviews) is essential [82,83], using frameworks, such as the technology acceptance model to understand user behavior [67,80].

### Data-Related Challenges

Data-related challenges include the following:

- Privacy and security: The collection and transmission of sensitive physiological data through smart textiles raise significant privacy and security concerns [84]. Robust data protection measures, secure data storage, and transparent data usage policies are essential to safeguard patient information and ensure compliance with relevant regulations [4,58,85]. Informed consent and authorized user access are paramount.
- Interoperability: The seamless integration of smart textile ECG data with existing EHRs and other health care systems is essential for efficient remote patient monitoring [64,86,87]. This requires consistent data formats, standard communication protocols, and a cloud-based architecture that ensures data exchange and interoperability across different service providers [88].
- Regulatory policy: The rapid evolution of smart textile technology necessitates a review and update of existing regulatory frameworks [86]. Clear standards and guidelines are needed to ensure safety and effectiveness, particularly concerning data privacy and security, in accordance with regulations like Health Insurance Portability and Accountability Act and Food and Drug Administration guidelines [89]. Transparent policies regarding data collection, usage, and sharing are crucial for building trust and facilitating adoption [90].

### Clinical Workflow Challenges

Clinical workflow challenges include the following:

- Communication and feedback: Effective communication and feedback between patients and health care providers are vital components of remote patient monitoring [91]. User-friendly clinical dashboards, timely alerts, and efficient reporting mechanisms are required to ensure timely intervention and improve clinical decision-making [64,73,74]. However, challenges include potential delays due to data loss, network issues, and data overload [8,70].
- Reimbursement: Clear and well‑defined reimbursement pathways are essential to ensure the financial viability of smart‑textile–based ECG monitoring within the health care system [74,76]. To support adoption, the cost‑effectiveness of these smart wearable systems must be demonstrated, and sustainable reimbursement models need to be developed, taking into account factors such as monitoring duration and clinical service provision [92,93]. Evidence from clinician surveys also indicates support for a shared‑cost approach, where expenses are distributed among patients, insurance providers, and government payers [94].
- Quality improvement: Integrating smart textile data into established clinical workflows enables continuous learning and improvement through feedback loops and data-driven insights [63,95-98]. This requires robust data analysis methods, the development of meaningful clinical indicators, and a strong focus on data-driven decision-making within the health care system. A structured approach to quality improvement, including infrastructural and organizational changes, is crucial for maximizing the benefits of smart textile technology.

### Limitations

This scoping review provides a current overview of e-textile–based ECG monitoring but has several important limitations. First, our search strategy did not explicitly include ECG-specific keywords (eg, “ECG,” “EKG,” “Electrocardiogram”), which may have restricted the retrieval of engineering-focused studies emphasizing signal fidelity, such as QRS detection algorithms. While cardiovascular disease terms (eg, “Arrhythmia,” “Atrial Fibrillation”) and monitoring concepts (eg, “Holter,” “ambulatory monitoring”) were expected to capture most relevant literature, omitting explicit ECG terms could have excluded technical performance studies that do not reference disease-specific terminology. Future reviews should incorporate ECG-related keywords and engineering databases to ensure comprehensive coverage of signal processing and hardware design research.

Second, the evidence base is dominated by feasibility and proof-of-concept studies, reflecting the early developmental stage of this technology. These studies often involve small sample sizes and controlled laboratory environments, limiting generalizability to real-world clinical settings. While such controlled conditions are valuable for assessing baseline performance, they may not fully capture usability and reliability under everyday conditions. Furthermore, the absence of standardized testing protocols for key metrics, such as signal quality, wearability, and durability, makes cross-study comparisons challenging. We did not conduct a formal risk of bias assessment, so the findings should be interpreted with caution.

Third, the scope of this review was primarily focused on signal quality, performance under static and dynamic conditions, and user experience. Wash durability, a critical factor for long-term usability, was outside the main scope. Future reviews should evaluate standardized laundering protocols and durability metrics, as these factors are essential for practical implementation and regulatory approval.

Despite these limitations, textile-based ECG monitoring demonstrates compelling potential benefits that conventional ECG methods cannot easily replicate. These include improved access to care for remote or underserved populations, continuous monitoring for the early detection of subtle cardiac changes, enhanced patient comfort and adherence due to increased wearability, and potential cost savings through earlier intervention. While further clinical validation and standardization are necessary, these advantages underscore the transformative potential of smart textiles in cardiac care.

### Conclusion

In conclusion, smart textiles offer the potential for the valuable long-term monitoring of ECG parameters, improving the detection of transient events and reducing intervention times. Further, their capacity for unobtrusive, remote monitoring and seamless integration with other health metrics offers unique advantages over conventional ECG. However, widespread support and adoption within clinical workflows depend on addressing the identified limitations, incorporating user feedback, and demonstrating the clinical utility and cost-effectiveness of this technology. Only with reliable and meaningful data can textile-based ECG monitoring serve as a valuable diagnostic tool to guide care, provide a more complete picture of heart health, and improve treatment decisions.

## Supplementary material

10.2196/74261 Multimedia Appendix 1 Database search details.

10.2196/74261 Checklist 1 PRISMA-ScR checklist.

## Abbreviations

AF: atrial fibrillation
ECG: electrocardiogram
EHR: electronic health record
MeSH: Medical Subject Headings
PRISMA: Preferred Reporting Items for Systematic Reviews and Meta-Analyses
