# Supplementary material for: Applications of Smart Textiles for Ambulatory Electrocardiogram Monitoring: Scoping Review of the Literature
Source: JMIR Cardio. 2026 Mar 2;10:e74261. doi: 10.2196/74261 (PMC12954708; doi:10.2196/74261)
Supplement: Multimedia Appendix 1 [file cardio-v10-e74261-s001.docx]

Database search details.

The literature search was conducted in PubMed/MEDLINE and EMBASE (via OVID). The strategy combined controlled vocabulary (MeSH/Emtree terms) and keywords to capture studies on textile-based ECG monitoring within cardiovascular care contexts. Three main concepts were used: (1) cardiovascular conditions, (2) smart textile technologies, and (3) monitoring and rehabilitation. Boolean operators were applied to combine these concepts, and limits were set for publication years (2000–present) and English language. The final search was executed in September 2025.

**1) PubMed/MEDLINE:**

(exp Cardiovascular Diseases OR arrhythmia OR atrial fibrillation OR heart disease OR cardiac disorders) AND (smart textile OR e-textile OR smart clothing OR textile electrode OR biosensor* OR wearable* OR wearable technolog*) AND (exp cardiac rehabilitation OR remote monitoring OR holter monitor* OR ambulatory monitoring OR cardiovascular rehab*) Limits: Humans, Publication Years 2000–2025

**2) EMBASE (via OVID):**

#1 ('exp cardiac rehabilitation' OR ('cardiac'/exp OR cardiac) AND ('rehabilitation'/exp OR rehabilitation) OR 'remote monitoring'/exp OR 'remote monitoring' OR (remote AND ('monitoring'/exp OR monitoring)) OR 'holter'/exp OR holter) AND monitor* OR 'ambulatory monitoring'/exp OR 'ambulatory monitoring' OR (ambulatory AND ('monitoring'/exp OR monitoring)) OR 'cardiovascular'/exp OR cardiovascular) AND rehab* AND [2000-2025]/py

#2 ('smart textile'/exp OR 'smart textile' OR (('smart'/exp OR smart) AND ('textile'/exp OR textile)) OR 'e textile'/exp OR 'e textile' OR 'smart clothing'/exp OR 'smart clothing' OR (('smart'/exp OR smart) AND ('clothing'/exp OR clothing)) OR 'textile electrode' OR (('textile'/exp OR textile) AND ('electrode'/exp OR electrode)) OR biosensor* OR wearable* OR wearable) AND technolog* AND [2000-2025]/py

#3 ('exp cardiovascular diseases' OR (exp AND ('cardiovascular'/exp OR cardiovascular) AND ('diseases'/exp OR diseases)) OR 'arrhythmia'/exp OR arrhythmia OR 'atrial fibrillation'/exp OR 'atrial fibrillation' OR (atrial AND ('fibrillation'/exp OR fibrillation)) OR 'heart disease'/exp OR 'heart disease' OR (('heart'/exp OR heart) AND ('disease'/exp OR disease)) OR 'cardiac disorders' OR (('cardiac'/exp OR cardiac) AND ('disorders'/exp OR disorders))) AND [2000-2025]/py

#4 #1 AND #2 AND #3
